# Stability response of alpine meadow communities to temperature and precipitation changes on the Northern Tibetan Plateau

**DOI:** 10.1002/ece3.8592

**Published:** 2022-02-16

**Authors:** Chunyu Wang, Junbang Wang, Fawei Zhang, Yongsheng Yang, Fanglin Luo, Yingnian Li, Jiexia Li

**Affiliations:** ^1^ Northwest Institute of Plateau Biology Chinese Academy of Science Xining China; ^2^ University of Chinese Academy of Sciences Beijing China; ^3^ Key Laboratory of Ecosystem Network Observation and Modeling Institute of Geographic Sciences and Natural Resources Research National Ecosystem Science Data Center Chinese Academy of Sciences Beijing China

**Keywords:** alpine meadow, biomass temporal stability, dominant functional group, functional groups asynchrony, temperature and precipitation

## Abstract

Biomass temporal stability plays a key role in maintaining sustainable ecosystem functions and services of grasslands, and climate change has exerted a profound impact on plant biomass. However, it remains unclear how the community biomass stability in alpine meadows responds to changes in some climate factors (e.g., temperature and precipitation). Long‐term field aboveground biomass monitoring was conducted in four alpine meadows (Haiyan [HY], Henan [HN], Gande [GD], and Qumalai [QML]) on the Qinghai‐Tibet Plateau. We found that climate factors and ecological factors together affected the community biomass stability and only the stability of HY had a significant decrease over the study period. The community biomass stability at each site was positively correlated with both the stability of the dominant functional group and functional groups asynchrony. The effect of dominant functional groups on community stability decreased with the increase of the effect of functional groups asynchrony on community stability and there may be a ‘trade‐off’ relationship between the effects of these two factors on community stability. Climatic factors directly or indirectly affect community biomass stability by influencing the stability of the dominant functional group or functional groups asynchrony. Air temperature and precipitation indirectly affected the community stability of HY and HN, but air temperature in the growing season and nongrowing season had direct negative and direct positive effects on the community stability of GD and QML, respectively. The underlying mechanisms varied between community composition and local climate conditions. Our findings highlighted the role of dominant functional group and functional groups asynchrony in maintaining community biomass stability in alpine meadows and we highlighted the importance of the environmental context when exploring the stability influence mechanism. Studies of community stability in alpine meadows along with different precipitation and temperature gradients are needed to improve our comprehensive understanding of the mechanisms controlling alpine meadow stability.

## Graphical abstract

Based on long‐term biomass monitoring data at four alpine meadow sites, we highlighted the importance of the dominant functional group and functional groups asynchrony for maintaining community biomass stability in alpine meadows and the importance of environmental context when exploring the stability influence mechanism.

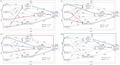

## 1 INTRODUCTION

Grasslands are the dominant landscape in much of China and account for 40% of the national land area (Kang et al., 2007). A stable grassland ecosystem is vital to ensure sustainable ecosystem functioning and human well‐being. Stability is a fundamental property of ecological systems, and temporal stability (defined as the inverse of temporal variability that measures the variation of ecological properties over time (Pimm, 1984)) is considered to be the most frequently measured aspect of stability in empirical studies (Huang et al., 2020). In particular, the temporal stability of community biomass production, and its regulatory mechanisms have received much recent attention in the context of global climate change (Xu et al., 2015).

In general, the stability of community biomass may depend on several ecological factors (e.g., community composition and interactions between species) and environmental factors (e.g., climate change, disturbance, and nutrient supply). In terms of community composition, dominant plant species in the community may affect ecosystem stability if they are well adapted to fluctuations in the availability of resources and can respond directly to climate change (Garcia‐Palacios et al., 2018). Some studies have found that the dominant species may strongly regulate the temporal stability of plant communities (Ma et al., 2017; Sasaki & Lauenroth, 2011; Zelikova et al., 2014). Species asynchrony, which generally means temporal asynchrony in the population dynamics of different species, is a common feature of ecological communities and may be attributed to asynchronous species responses to environmental fluctuations (Gonzalez & Loreau, 2009; Ma et al., 2017). Studies have reported that community stability seems to arise from compensatory interactions among major components at both the species and functional group levels (Bai et al., 2004), and that species richness and species asynchrony promote community stability (Zhang, Loreau, et al., 2018). Furthermore, in the response to environmental changes, species from different functional groups are likely to diverge, while species within a functional group converge, likely causing asynchrony among functional groups (Diaz & Cabido, 2001; Huang et al., 2020). Long‐term experimental observation of alpine meadows has demonstrated that plant functional group asynchrony maintains community biomass stability (Zhou et al., 2019). Therefore, further consideration of functional groups asynchrony can improve our understanding of community biomass stability.

Temperature and precipitation were found to be the key climatic drivers of ecosystem processes and the main driving forces for grassland community change (Liu et al., 2015; Wu et al., 2011). Much of the earth is experiencing climate warming and changes in precipitation patterns, and climate has already altered the function, composition, and structure of many ecosystems, with potentially large influences on plant community dynamics (Ma et al., 2017; Verrall & Pickering, 2020). A study based on long‐term datasets of grassland species composition at nine grassland sites in North America found that compensatory dynamics will become more important to the stability of sites that experience increased precipitation variability (Hallett et al., 2014). Field experiments on temperate steppe sites have shown that an increase in growing season precipitation enhances plant species richness, thus promoting plant biomass temporal stability, while daytime warming lowers community temporal stability by reducing the abundance of dominant, stable species (Xu et al., 2015; Yang et al., 2017). However, a long‐term monitoring study of alpine meadows suggested that neither asymmetrical daytime and night‐time warming, nor precipitation, has a significant effect on community temporal stability. Field‐based climate manipulation experiments suggested that warming lowers the biomass temporal stability of alpine meadows by reducing the degree of species asynchrony (Huang et al., 2020; Ma et al., 2017). However, the controversy surrounding the effects of climate change on community temporal stability has arisen because these findings were site‐specific and method‐specific. These uncertainties and controversies motivate us to verify the climatic effect on community temporal stability, based on more sites.

The Qinghai‐Tibet Plateau (QTP), known as “the third pole”, has experienced ubiquitous, rapid warming in the past several decades, and has become slightly wetter, albeit with a high inter‐annual variation of precipitation. Grasslands on the QTP are sensitive and vulnerable to climate change, because of low temperatures and cryophilic vegetation (Li, Wu, et al., 2020). Alpine meadows in China, a typical vegetation type on the QTP, cover approximately 637,000 km^2^. Climate change has exerted a profound impact on plant biomass and may affect the biomass temporal stability of alpine grasslands (Fu et al., 2019; Huang et al., 2020; Li et al., 2019; Ma et al., 2017; Zhang et al., 2018). In particular, the warming rate and precipitation trend show obvious seasonal differences (Chen et al., 2013). Examining the biomass temporal stability of alpine meadows under varying climate conditions is critically important for exploring the stability influence mechanism and maintaining service functions for alpine meadow ecosystems on the QTP. Several studies have demonstrated that climate warming and precipitation change have, or may have, impacts on community biomass stability (Huang et al., 2020; Ma et al., 2017). However, due to high spatial heterogeneity, the characteristics of climate change and the composition of vegetation communities on the QTP are complex and diverse. We know little about whether ecosystem productivity stability and the associated climatic mechanism are specific to particular communities and climates. Given that changes in temperature and precipitation in the growing season and non‐growing season may have different effects on grassland productivity and temporal stability (Fu et al., 2019; Huang et al., 2020), the changes in temperature and precipitation in both these seasons should be considered in the study of biomass stability for alpine grasslands. Moreover, most studies were based on short‐term climate manipulation experiments, which may introduce bias in predicting the temporal responses of communities to future climate change (Andresen et al., 2016; Zhou et al., 2019); consequently, research based on a single site has great uncertainty for exploring the general mechanism of alpine meadow stability. Long‐term studies of natural alpine grassland ecosystems among different sites are still scarce. To fill these knowledge gaps, we collected a long‐term field dataset from 4 typical alpine meadow sites on the QTP to address the following questions: (1) What are the trends for biomass and stability at the community and functional group levels at the four sites? (2) Are there any effects of air temperature and precipitation in the growing season and non‐growing season on the temporal stability of community biomass? (3) How do climate factors and ecological factors influence the stability in different alpine meadow communities?

## 2 MATERIALS AND METHODS

### 2.1 Study area and experimental design

**FIGURE 1:**
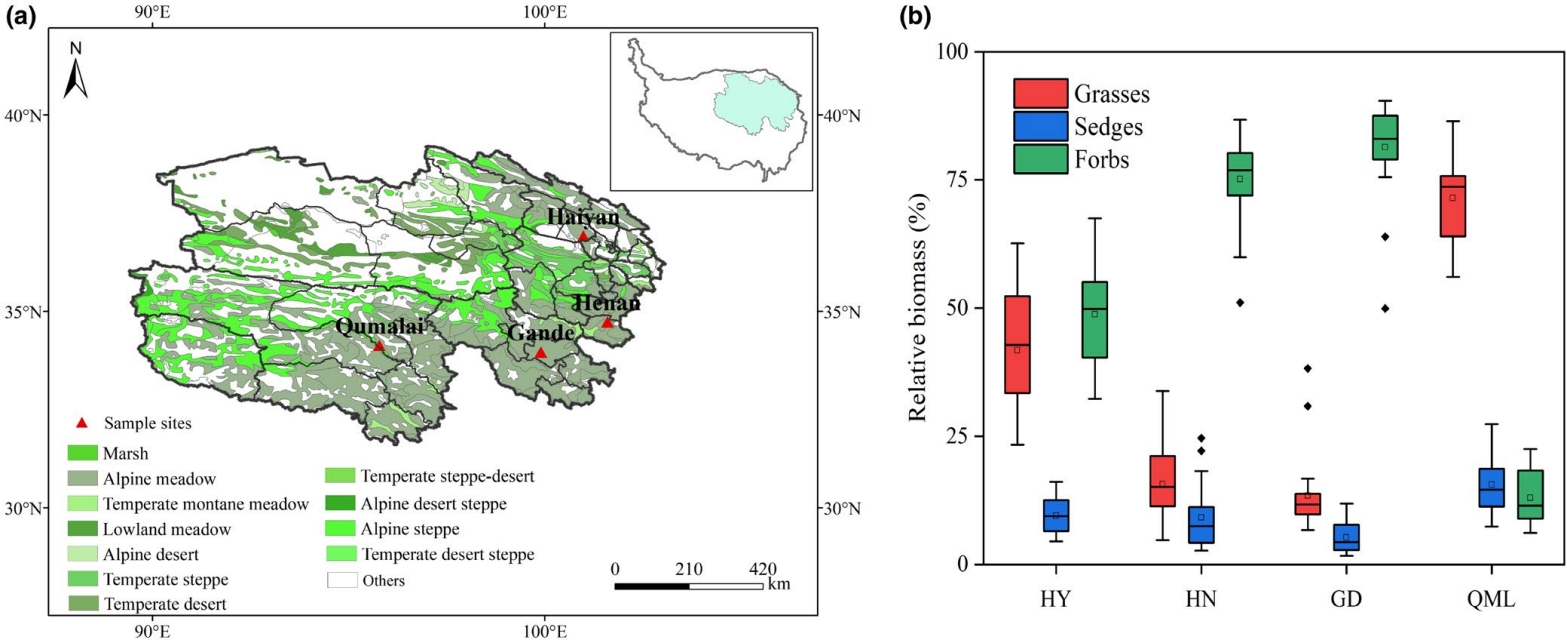
Geographical location of the study sites (a) and relative biomass of functional groups at Haiyan (HY), Henan (HN), Gande (GD), and Qumalai (QML) (b). The horizontal black line and the black box within each box represent the median and the mean, respectively. The lower and upper edges of the bars outside the boxes represent the 25th and 75th percentiles, and the 5th and 95th percentiles of all data, respectively. Outliers are represented by small dots beyond the 5th and 95th percentile bars

Long‐term monitoring data for natural alpine grassland ecosystems are scarce. However, we conducted an experimental study for about 20 years to monitor the aboveground biomass of four alpine meadow sites in Qinghai Province, northeastern Tibetan Plateau (Figure 1a). The four study sites, namely Haiyan (HY), Henan (HN), Gande (GD), and Qumalai (QML), are all near the meteorological stations. The location, environmental information, and the study period of the four sites are shown in Figure 1a and Table 1. The four alpine meadow sites are covered by mesic meadows consisting of C3 perennial species and the soil types are meadow soil. The growing season lasts from May to September and herbaceous plants on alpine grasslands usually start to sprout in May, peak at their maximum coverage and aboveground biomass in August and senesce in early September. There is a gradient with respect to altitude and annual mean temperature (AMT) for the four sites. The AMTs at HY (1997–2015), HN (1994–2015), QML (1994–2015), and GD (2001–2013) were 1.39, 0.19, −1.27 and −1.85°C, respectively. The average annual mean precipitation (AMP) was 413.16, 559.63, 430.28, and 530.66 mm, respectively, with precipitation mainly concentrated in the rainy season (May–September). To compare the degree of climate dryness, we calculated the aridity index (AI) (Sun et al., 2020) (AI = AMP/(AMT +10)). The average values of AI showed that GD was the wettest, followed by HN, QML, and HY (driest).

**TABLE 1:** Location and environmental information for study sites in the alpine meadows of Qinghai, China

Site	Latitude (N)	Longitude (E)	Elevation (m)	AMT (°C)	AMP (mm)	Constructive plants/dominant plants	Study period
HY	36°57′01″	100°54′21″	3088	0.6 to 1.9	248.2–504.2	*Poa crymophila; Stipa purpurea; Kobresia humilis; Artemisia scoparia*	1997–2015
HN	34°44′03″	101°36′04″	3500	−1.1 to 0.9	384.1–733.3	*Elymus nutans Griseb*.; *Double*‐*stigma Bulrush*; *Puccinellia tenuiflora* *(Griseb*.*) Scribn*.; *Kobresia pygmaea*	1994–2015
GD	33°57′57″	99°52′14″	4110	−3.9 to −1.0	418.6–667.3	*Elymus nutans Griseb.; Koeleria macrantha; Festuca ovina L.; Kobresia humilis*	1994–2015
QML	34°07′12″	95°48′06″	4175	−1.9 to −0.2	329.3–566.4	*Festuca ovina L*.; *Kobresia pygmaea*; *Poa crymophila*; *Carex* spp.	2001–2013

Abbreviations: AMP, annual mean precipitation; AMT, annual mean temperature; GD, Gande; HN, Henan; HY, Haiyan; QML, Qumalai.

The experimental plots for monitoring aboveground biomass had an area of 100 m × 100 m enclosed by an animal husbandry meteorological station at all four sites, and the areas were equally divided into four 50 m × 50 m blocks. The aboveground biomass (AGB) of each site was harvested and measured monthly in May–September of each year from three subplots of size 1 m × 1 m in each sample plot randomly distributed to contain less vegetation heterogeneity. Plants were clipped at ground level in the quadrat and divided into three functional groups (grasses, forbs, and sedges). All plants were weighed after oven‐drying at 65°C to constant weight. The sum of the biomass in the three functional groups was the community biomass. As the maximum AGB usually occurs in August (the peak of the growing season), we defined annual biomass production as the aboveground biomass observed in August. Climatic data at these 4 sites, including monthly mean temperature and precipitation, were derived from the local meteorological station. Data were subjected to a preliminary quality check, and according to the phenological characteristics of alpine grassland, we calculated the mean temperature and precipitation of the growing season (May–August) (GST, GSP) and non‐growing season (October in the year before the study year to April in the study year) (NGST, NGSP).

### 2.2 Methods

1. According to the average relative biomass (the ratio of functional group biomass to total community biomass) of each site in the study, the functional group with the highest relative biomass was defined as the dominant functional group, and the functional group next to the dominant functional group was defined as the subdominant functional group. The dominant functional groups at HY, HN, and GD were forbs, and the subdominant functional groups were grasses. The dominant and subdominant functional groups at QML were grasses and sedges, respectively (Figure 1b).
2. The biomass temporal stability of the community and functional groups was measured as the ratio of mean biomass (m) to its temporal standard deviation (s) (Lehman & Tilman, 2000) at each site over the study period. The degree of functional groups asynchrony was quantified by the community‐wide asynchrony index (Loreau & Mazancourt, 2008), defined as:

$$1‐\alpha_{x}=1‐\sigma^{2}/{\left(\sum_{i=1}^{S}\sigma_{i}\right)}^{2}$$

where $\alpha_{x}$ is functional groups synchrony,$\sigma^{2}$ is the variance of community biomass, and $\sigma_{i}$ is the standard deviation of biomass of functional group *i* in a plot with *S* functional groups.

Since most stability studies have been based on different samples in a study area, which could not reflect the temporal dynamics of stability, we used the moving window method to explore the changes of stability at a given time scale. Many related experimental studies used 5 years as the time scale (Hautier et al., 2014; Ma et al., 2017); therefore, we calculated the biomass stability and functional groups asynchrony with a window size of 5 years.

3. We constructed piecewise structural equation modeling (piecewiseSEM) to examine how climate and ecological factors and their interaction influence community biomass stability through direct or indirect pathways. This structural equation modeling can circumvent restrictions of sample size (Shipley, 2000). The *p*‐value of Fisher's C statistic was used to test the rationality of the structure of piecewiseSEM. Fisher's C statistic and AIC were used to evaluate the overall goodness of model fit. The piecewiseSEMs were constructed based on a priori knowledge from the literature (Zhou et al., 2019) and the causal relationships found in multiple regression (Table 2) and correlation analysis (Figure S1) of effects on community biomass stability. The paths of SEMs were modified according to *p* values of Fisher's C statistic and AIC (Luo et al., 2021). The stability of nondominant functional groups was not considered in the SEM, as it did not effectively improve the fit of the model. To better identify and compare the influence mechanism of climate factors and ecological factors on the biomass temporal stability of different alpine meadow communities, similar structures were used in the piecewiseSEMs for the four sites. We reported the standardized coefficient for each path and the proportion of variance (*R* ^2^) explained for each dependent variable in the models. Note that the climate data inputted into the SEMs were 5‐year averages for the same moving window. Simple linear regressions and correlation analyses were used to assess the trend of the study object and the correlation relationship among the factors, respectively. Multiple linear regression was used to identify environmental and ecological factors impacting biomass temporal stability of the alpine grassland community. Analyses were carried out using R software (version 3.6.2; R Core Team, 2015; https://www.R‐project.org) and MATLAB 2015b (version 8.6.0.267246), and the SEMs were constructed using the ‘piecewiseSEM’ package in R software (Lefcheck, 2016).

**TABLE 2:** The parameters of multiple linear regression included in predicting community biomass stability and environmental and biological factors

	Standard coefficient	Pr(>|t|)	Multiple *R*‐squared	*F*‐statistic	*p*‐value
Site	0.521	0.662	0.783	20.87 on 9 and 52 DF	2.475e^−11^
GST	1.635	0.135			
NGST	−2.395	0.023*			
GSP	0.001	0.905			
NGSP	−0.033	0.347			
Functional groups asynchrony	16.502	2.17e^−11^***			
Stability of grasses	0.668	0.023*			
Stability of forbs	0.498	0.002**			
Stability of sedges	0.581	0.196			

Note

Asterisks indicate statistical significance (***p < .001; **p < .01; *p < .05).

Abbreviations: DF, degree of freedom; GSP, growing season precipitation; GST, growing season temperature; NGSP, non‐growing season precipitation; NGST, non‐growing season temperature.

## 3 RESULTS

### 3.1 Changes in climate and aboveground biomass

**FIGURE 2:**
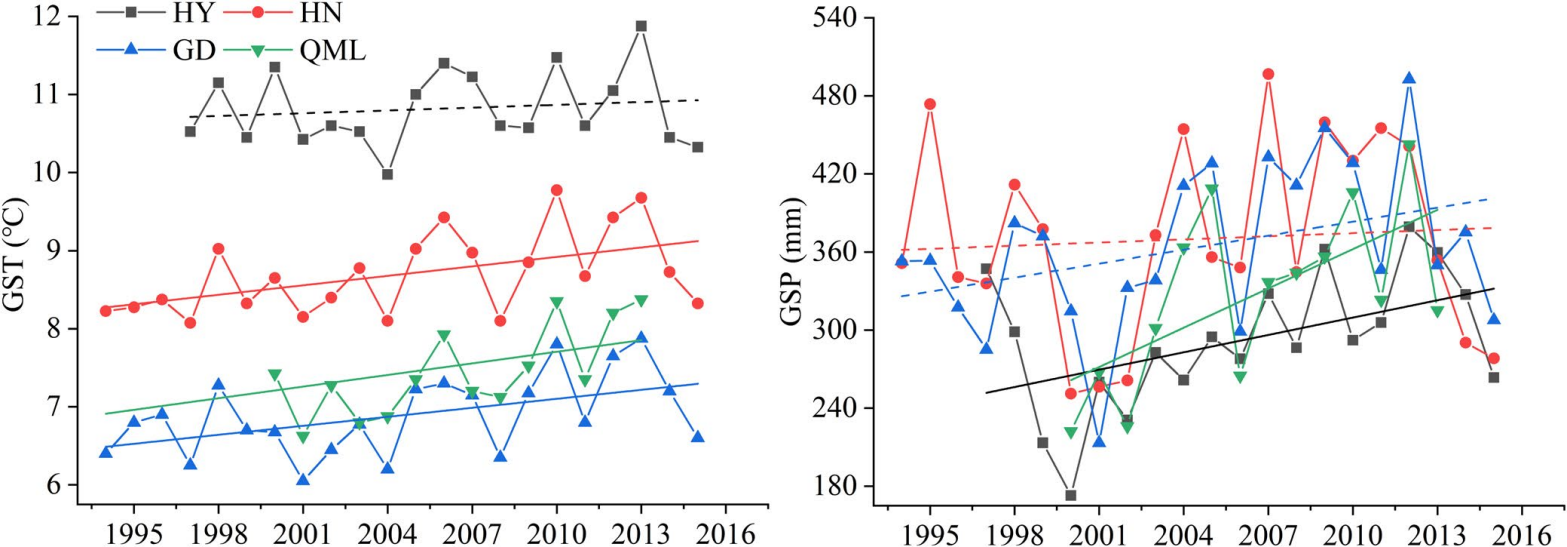
Trends of temperature (GST) and precipitation (GSP) in the growing season at Haiyan (HY), Henan (HN), Gande (GD), and Qumalai (QML) during the study period. The fitted lines were determined by a linear regression model. Solid lines denote the relationship with *p* < .05 and dashed lines denote the relationship with *p* ≥ .05

In the past 20 years, the annual mean temperature at HN, GD, and QML increased significantly, while the increasing trend at HY was not significant. The GST at HY showed no significant trend, while that at the other three sites showed significant upward trends (Figure 2). Notably, the rate of interannual warming in the non‐growing season was higher than that in the growing season. For precipitation, GSP showed significant increases at HY and QML (4.45 and 10.07 mm·year^−1^, respectively). In general, trends toward a warmer, moister growing season climate were observed at HY and QML, and just significant warming trends were observed at HN and GD.

**FIGURE 3:**
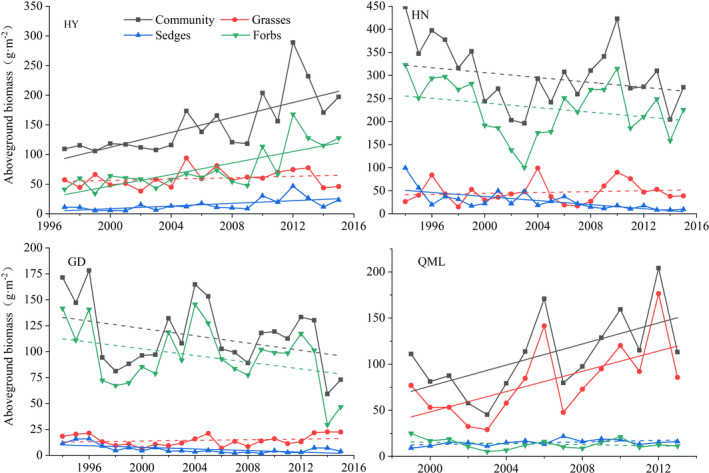
Trends in aboveground biomass at Haiyan (HY), Henan (HN), Gande (GD), and Qumalai (QML) during the study period. The fitted lines were determined by a linear regression model. Solid lines denote the relationship with *p* < .05 and dashed lines denote the relationship with *p* ≥ .05

The highest and lowest average community biomass occurred at HN (303.0 g·m^−2^) and QML (109.6 g·m^−2^), respectively. Community biomass at HN and GD presented a statistically nonsignificant decreasing trend, while that at HY and QML displayed a significant increasing trend (6.29 and 5.87 g·m^−2^ year^−1^, respectively) (Figure 3). For functional groups, sedges and forbs at HY showed stronger interannual variability and presented increasing trends (1.12 and 4.85 g·m^−2^ year^−1^, respectively). The temporal trends in biomass of each functional group at HN and GD were similar: the sedges displayed significant decreasing trends (2.21 and 0.39 g·m^−2^ per year, respectively). At QML, the biomass of grasses significantly increased, at a rate of 5.14 g·m^−2^ per year.

### 3.2 Biomass temporal stability and plant functional groups asynchrony

**FIGURE 4:**
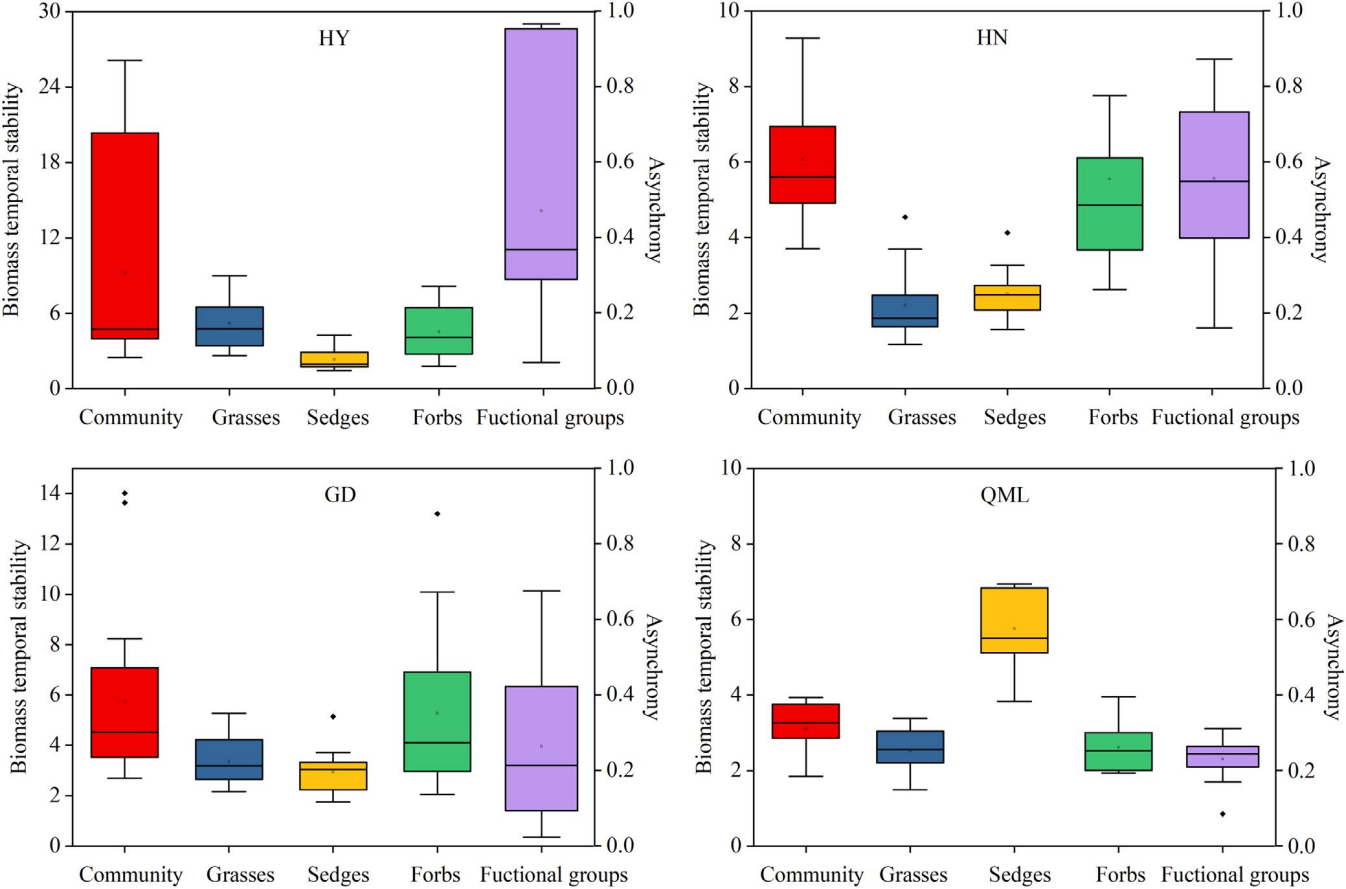
Average biomass temporal stability and functional groups asynchrony at Haiyan (HY), Henan (HN), Gande (GD), and Qumalai (QML). The horizontal black line and the black box within each box stand for the median and the mean, respectively. The lower and upper edges of the bars outside the boxes represent the 25th and 75th percentiles, and the 5th and 95th percentiles of all data, respectively. Outliers are represented by small dots beyond the 5th and 95th percentile bars

The average stability of the communities and functional groups, as well as functional groups asynchrony, were analyzed with a 5‐year moving window (Figure 4). The highest average community stability was found in HY and the highest functional groups asynchrony was found at HN. Biomass stability generally increased along the hierarchy of organizational level (from functional groups to community). At the functional groups level, the stability of dominant and subdominant functional groups was generally higher than that of the nondominant functional group.

**FIGURE 5:**
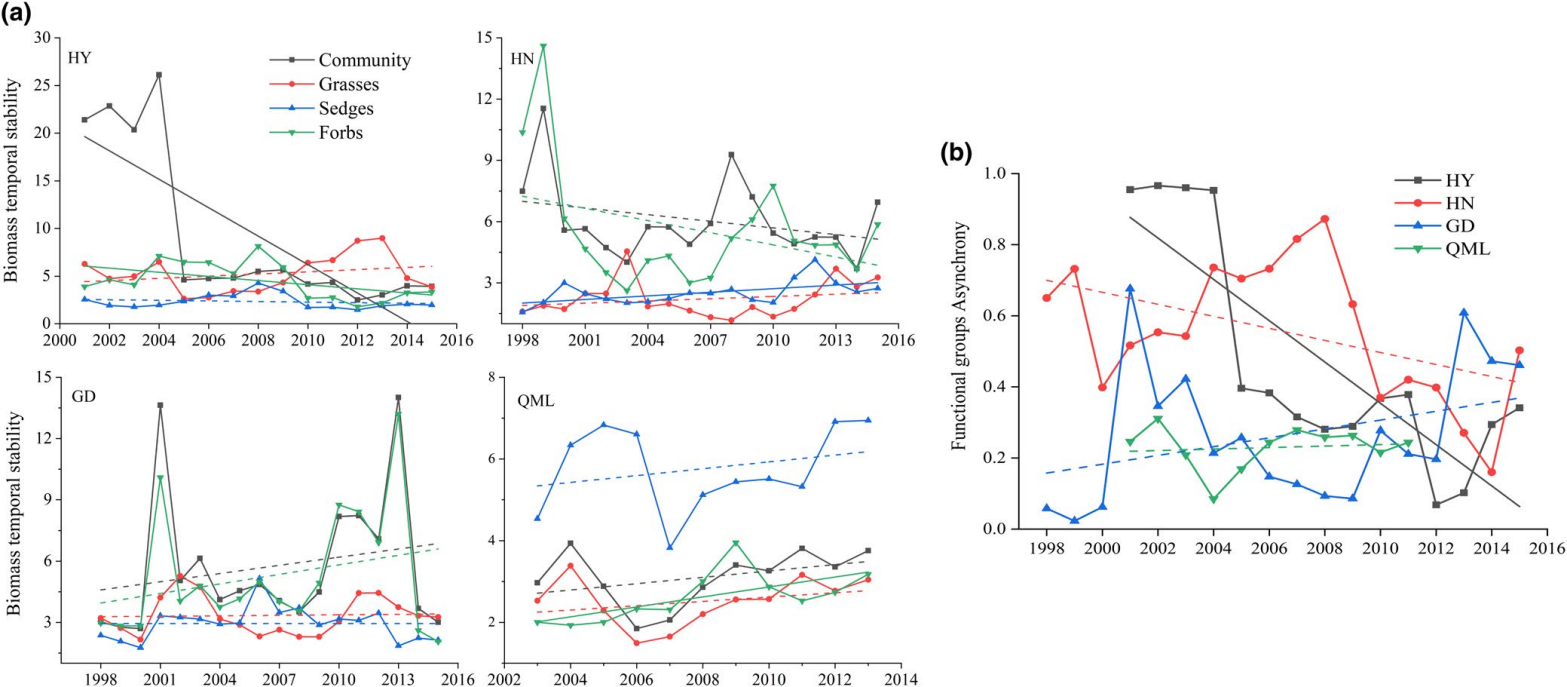
Trends in the average biomass temporal stability (a) and functional groups asynchrony (b) at Haiyan (HY), Henan (HN), Gande (GD), and Qumalai (QML) during the study period, based on a 5‐year moving window. The fitted lines were determined by a linear regression model. Solid lines denote the relationship with *p* < .05, and dashed lines denote the relationship with *p* ≥ .05

We next analyzed the changes in biomass stability and functional groups asynchrony (Figure 5). At the community level, only the stability at HY showed a significant (*p* < .01) decreasing trend. The decreasing trend at HN, and the increasing trends at GD and QML, are not statistically significant. At the functional group level, the stability of forbs at HY displayed a significant decreasing trend, and the stability of sedges at HN significantly increased. The functional groups asynchrony at HY presented a significant (*p* < .01) decreasing trend, while that at HN displayed a nonsignificant decreasing trend. The functional groups asynchrony at GD and QML showed a nonsignificant increasing trend.

### 3.3 The relationships among climate factors, ecological factors, and community biomass stability

Multiple linear regressions, based on moving windows, were constructed with the community stability of four sites as the criterion variable; and site, GST, NGST, NGST, GSP, NGSP, functional groups asynchrony, and stability of functional groups as predictor variables. The result (Table 2) was that community temporal stability was positively correlated with functional groups asynchrony, stability of grasses and forbs, and NGST, but there were no significant relationships between community stability and site, GST, GSP, and NGSP.

Correlation analyses were used to assess how environmental and ecological factors related to community stability and the interactions between biotic and abiotic factors for each site. Correlation analyses showed that community stability at all sites was significantly (*p* < .05) positively correlated with functional groups asynchrony (Figure S1). However, only the community stability in HY was significantly correlated with GST and GSP, and the community stability of other sites had no significant correlation with climate factors. Moreover, the factors influencing each functional group stability and functional group asynchrony of the four sites were also different. Therefore, we hypothesized that climate factors may affect the community stability of the four sites in different ways, in both direct and indirect ways.

### 3.4 The effects of environmental factors and ecological factors on community biomass temporal stability

**FIGURE 6:**
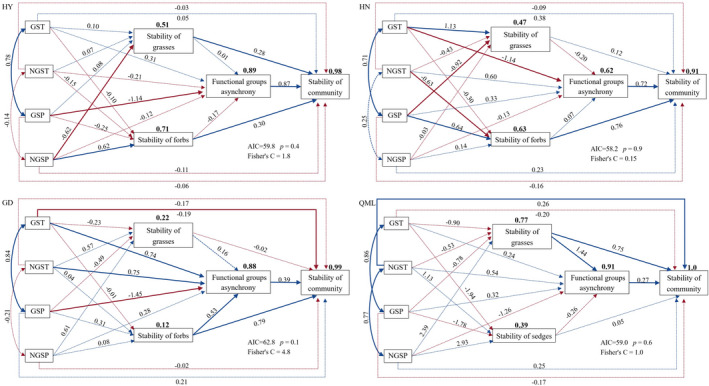
The structural equation models of environmental and ecological factors on biomass temporal stability at Haiyan (HY), Henan (HN), Gande (GD), and Qumalai (QML). The numbers above the endogenous variables represent the proportion of variance explained for each dependent variable in the model (R^2^). The solid and dashed lines represent significant (*p* < .05) and nonsignificant (*p* ≥ .05) effects, respectively. The numbers on the one‐way arrows are the standard coefficients, and numbers on the two‐way arrows are the standard correlation coefficients. Blue and red arrows represent positive and negative pathways, respectively. GSP, growing season precipitation; GST, growing season temperature; NGSP, non‐growing season precipitation; NGST, non‐growing season temperature

The *p* values of Fisher's C statistic for all four sites were above 0.05, indicating that the structures of models were reasonable. The SEM path analysis explained the change in community stability, with a high *R* ^2^ (*R* ^2^ > 90%); the *R* ^2^ of functional groups asynchrony also exceeded 60%. Consistent with the results of multiple linear regression, SEMs for the four sites revealed that both the stability of the dominant functional group and functional groups asynchrony had a direct positive effect on community biomass stability (Figure 6). Climate factors, directly and indirectly, affect the stability of community biomass. At HY and HN, climate factors indirectly affected community stability by increasing or decreasing the stability of dominant and subdominant functional groups, as well as the functional groups asynchrony. However, at GD, climate factors not only directly affected community stability but also indirectly affected stability through their impacts on functional groups asynchrony. At QML, with grasses as the dominant functional group, NGST had a directly positive effect on community stability. Considering the total effects of climate factors on community stability, the air temperature had no significant effect on the community stability of HY, while GSP and NGSP had, respectively, negative (−0.99) and positive (0.02) effects on community biomass stability (Table 3). At HN, the temperature had a negative effect, while precipitation had a positive effect on community stability, and the effect of temperature was greater than that of precipitation regardless of the growing/non‐growing season. However, both GST and NGST had positive effects on the community stability of GD, but only the precipitation in the growing season had a negative (−0.57) effect on community stability. Precipitation of QML had no significant effect on community stability, and only the NGST had a positive (0.26) effect on community stability. Moreover, an interesting finding from the four sites was that the effect of dominant functional groups on community stability decreased with the increase of the effect of functional groups asynchrony on community stability.

**TABLE 3:** Standardized total effects of environmental and ecological factors on community biomass stability based on statistically significant paths in the SEMs

	GST	NGST	GSP	NGSP	Functional groups asynchrony	Stability of grasses	Stability of forbs	Stability of sedges
HY	–	–	−0.99	0.02	0.87	0.28	0.30	–
HN	−0.82	−0.48	0.49	0.02	0.72	–	0.76	–
GD	0.12	0.29	−0.57	–	0.39	–	1.0	–
QML	–	0.26	–	–	0.27	1.14	–	–

Abbreviations: GD, Gande; GSP, growing season precipitation; GST, growing season temperature; HN, Henan; HY, Haiyan; NGSP, non‐growing season precipitation; NGST, non‐growing season temperature; QML, Qumalai.

## 4 DISCUSSION

Using long‐term observational field data from four alpine meadow communities on the northern QTP, we analyzed the changes in aboveground biomass and stability of alpine meadow communities over 20 years, and we investigated the mechanism of community stability. We found that the stability of dominant functional groups and functional groups asynchrony played an important role in maintaining the community biomass stability of alpine meadows and that there was a trade‐off relationship between the effects of these two factors on community temporal stability. Climate factors directly or indirectly affected community biomass stability. The mechanism underlying the effects of climate factors on community stability varied with the climate conditions and community composition of alpine meadows. More details of the mechanisms underlying changes in biomass and stability will be discussed below.

### 4.1 Changes in aboveground biomass of community and functional groups

Plants adapt and respond to climate change differently, depending on species and life‐forms (Byrne et al., 2017). The warmer and wetter trend enhanced the aboveground biomass of communities at HY and QML (Figure S2), and the increasing rate tended to be higher in the relatively dry and warm region (HY). This finding is consistent with Han et al. (2018). Different from HY and QML, the biomass of communities at HN and GD showed nonsignificantly decreasing trends with climate warming and the biomass of sedges showed significant decreasing trends. Although the climate at HN and GD is relatively humid, continued warming may lead to a reduction in soil water content. Shallow‐rooted sedges need to improve their water use efficiency by reducing their biomass to adapt to a water‐stressed environment under climate warming (Hu et al., 2008). With the increase of GSP during the study period, the biomass of forbs at HY showed a significant increasing trend, while that of grasses increased nonsignificantly. This was because the plant functional groups differ in their responses to precipitation change, with forbs benefiting more than grasses from increased water availability (Chelli et al., 2016). There was a significant increasing trend in the biomass of grasses at QML while that of forbs showed no significant change. This may reflect a combined response of grasses to the increase of GSP and GST, and the competition with forbs.

### 4.2 Response of functional group stability to temperature and precipitation changes

We found that the stability of the dominant and subdominant functional groups was generally higher than that of the nondominant functional group, similar to Ma et al. (2017) who found that the temporal stability of dominant species was greater than that of less abundant species. The effect of air temperature and precipitation on the stability of alpine meadow functional groups varied with the types of functional groups and climatic conditions. The SEM showed that the stability of grasses and forbs at HY had the same sensitivity to the change of NGSP, but grasses had a negative response and forbs had a positive response. The opposite response of the stability of grasses and forbs to NGSP may be due to grasses having a higher demand for heat at the beginning of the growing season, compared with the soil water demand of forbs. The frozen soil water, and snowpack that accumulated during the non‐growing season, could increase the soil moisture content (Nandintsetseg & Shinoda, 2011; Richardson et al., 2013), promoting the greening up and growth of forbs in the following spring and summer, and conductive to reducing biomass fluctuations. On the other hand, the greening up of grasses would be inhibited, thus causing fluctuation of biomass due to the increase of NGSP, which in turn would reduce the soil temperature. Similarly, the stability of forbs at HN was negatively affected by NGST and positively affected by GSP, and that of grasses was positively affected by GST, but negatively affected by GSP. Nevertheless, the SEM showed that neither warming nor changes in precipitation significantly affected the stability of dominant and subdominant functional groups at GD and QML; perhaps this indicates that the dominant and subdominant functional groups at the two sites are adapted to the local climate. The physiological responses of forbs, which accounted for more than 80% of the community biomass at GD, showed signatures of adaptation to resource‐constrained conditions and showed more adaptive strategies in their relationship with other plants (Zhang et al., 2020). Grasses and sedges contributed to approximately 87% of the community aboveground biomass at QML, which were relatively insensitive to perturbations due to their strong resource‐acquiring ability. In particular, grasses are generally extremely drought‐resistant and insensitive to precipitation (Ma et al., 2017).

### 4.3 Response of functional groups asynchrony to temperature and precipitation changes

The asynchrony of components in an intact community reflects the ecological response to meteorological fluctuations, that is, the combined effects of the physiological response and of competition (Lepš et al., 2019). The compensatory dynamics between species or functional groups can be driven by a variety of mechanisms (Gonzalez & Loreau, 2009; Grman et al., 2010). In our study, the SEM analysis showed that functional groups asynchrony at HY was negatively affected by GSP. Correlation analysis showed that GSP was positively correlated with the aboveground biomass of grasses, sedges (*p* < .05) and forbs at the relatively arid HY (Figure S2). The increase of GSP relieved the limitation of water on plant growth, resulting in the simultaneous increase of biomass in the three functional groups and decreasing the functional groups asynchrony, which further led to a decrease in community stability. A similar study (Zhou et al., 2019) was conducted at HY, which showed that precipitation did not significantly affect the plant functional groups asynchrony. The difference might be attributed to the different scales of precipitation selected as a climate factor. At the relatively humid HN, adequate heat can satisfy the growth requirements of plants. The increase of GST would promote synchronous changes of biomass in dominant and subdominant functional groups (grasses and forbs) and have a negative effect on the asynchrony of functional groups. The SEM for GD showed that functional groups asynchrony at GD was positively correlated with GST and NGST, but negatively correlated with GSP, and the positive effect may have offset the negative effect to some extent, leading to a nonsignificant trend of functional groups asynchrony. Different from the other three sites, the functional groups asynchrony at QML was not significantly affected by climate factors but was affected by the stability of dominant functional groups. These findings further illustrate the different sensitivities of functional groups asynchrony of different alpine meadow ecosystems to climate change, which may be attributed to the differences in climate conditions and community assemblages of plant functional groups and traits.

### 4.4 Mechanism of community biomass stability

Both climate and biological factors affected the community biomass stability of alpine meadows. Many studies have suggested that dominant species, dominant functional groups, or asynchrony of species and functional groups played a role in maintaining grassland community stability (Bai et al., 2004; Grman et al., 2010; Hallett et al., 2014; Hector et al., 2010; Li et al., 2018; Sasaki & Lauenroth, 2011; Yang et al., 2016; Zhou et al., 2019). However, our results based on four alpine meadow sites demonstrated that functional groups asynchrony and the stability of the dominant functional group were important mechanisms for maintaining community temporal stability. Notably, we found that the effect of functional group asynchrony on community stability decreased as the effect of stability of the dominant functional group increased (Table 2); that is, there may be a ‘trade‐off’ relationship between the effects of the two factors on community stability. Different attributes of the community play a role in regulating the temporal stability of an alpine ecosystem in the face of environmental changes. Functional groups asynchrony is expected to be more important in communities with a relatively even distribution of biomass among the constituent functional groups, whereas the traits of the dominant functional group are likely to be critical for stability in communities with an uneven biomass distribution. This phenomenon also occurs at the species level (De Boeck et al., 2018). One possible explanation is that competitive advantages of dominant species might be obstacles for the settlement and survival of other less competitive species (Li, Zhang, et al., 2020), thus affecting the interspecific relationships. Functional groups asynchrony and the strength and relative importance of compensatory dynamics may vary widely across communities, and changes may be driven by the environment (Grman et al., 2010). Moreover, increases in the strength of one stabilizing mechanism could compensate for losses in another, driven by complex changes in community structure, composition, and diversity (Grman et al., 2010). While needing evaluation with more experiments, this finding indicated that the changes in the dominant functional group deserve great attention when managing communities for the maintenance and temporal stability of ecosystem function (Shi et al., 2016).

The SEMs results showed that climate factors directly or indirectly affected the community biomass stability. The effect of precipitation changes during the growing season on community stability was generally greater than that in the non‐growing season. Moreover, the effects of climate factors on community stability varied with its own climatic condition and community composition. For the standardized total effects of climate factors in piecewiseSEM, the air temperature had no significant effect on community stability at HY, while precipitation had significant effects on community stability, and the negative impact of GSP was greater than that of NGSP. A previous study has reported that increasing GSP promoted biomass temporal stability by enhancing plant species richness (Xu et al., 2015). The different results between the two studies may be attributed to different climate conditions of the study sites, that is, the precipitation in Xu's study was 1.5 times that at HY. Environmental changes may modify the role of species interactions on community dynamics. Species or functional groups differ in their ecological attributes; therefore, they may respond differently to changes in abiotic environmental conditions (Kari & Ørjan, 2008). The degree and direction of the change in stability depended substantially on the nature of the environmental perturbation imposed on the community and on the responding functional groups. The net outcome of community stability is the combined effects of species interactions, and the environmental conditions experienced by the community (Kari & Ørjan, 2008). There were variations in environmental and community composition between the four sites in this study. Meanwhile, plants in some communities may have been environmentally selected to evolve specific functional traits to adapt to environmental conditions. Therefore, the degree and direction of the effect of climate factors on community stability varied with the environmental conditions and community composition.

**FIGURE 7:**
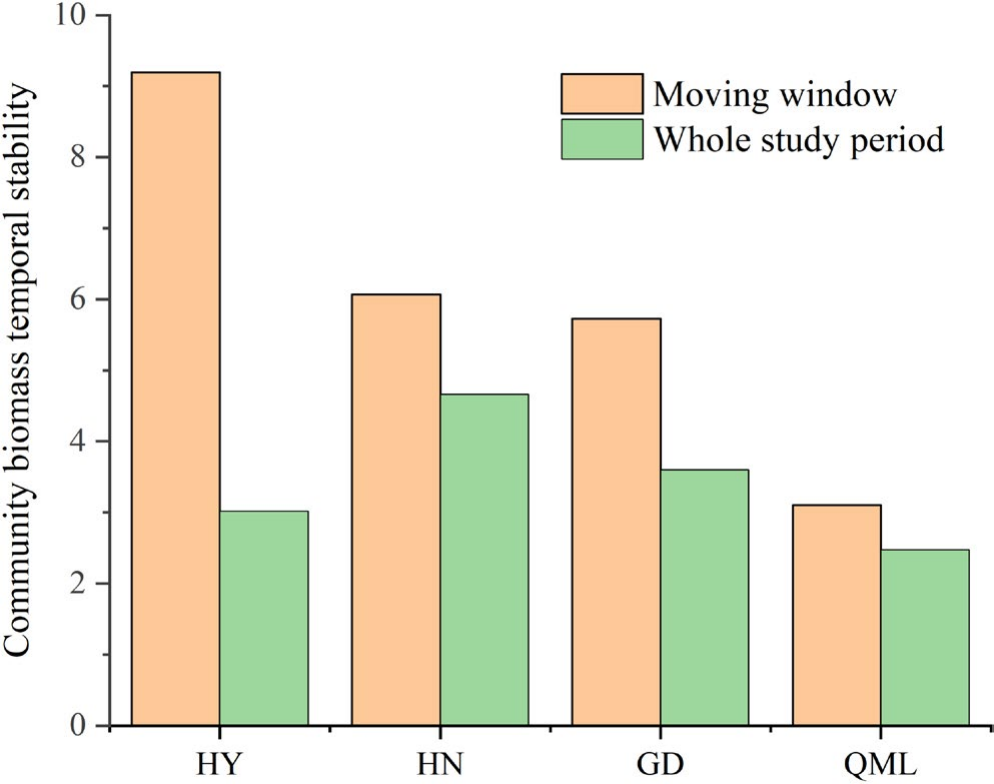
The community biomass stability of the four sites (Haiyan (HY), Henan (HN), Gande (GD), and Qumalai (QML)) throughout the study period and the average community stability based on moving window

Climate change will likely be multi‐faceted at a region (Shi et al., 2016). Since this study was based on long‐term observation experiments rather than climate manipulation experiments, the possibility of other nonmutually exclusive mechanisms could not be ruled out, such as atmospheric CO_2_ concentration and atmospheric nitrogen (N) deposition. A large field experiment conducted in mixed‐grass prairie in southeastern Wyoming showed that community evenness increased as dominant species decreased in biomass under elevated CO_2_, which led to the alteration of community dominance patterns and the increase in production stability (Zelikova et al., 2014). Fertilization, intentional or inadvertent via atmospheric nitrogen (N) deposition significantly affects the structure of natural grassland communities (Grman et al., 2010; Wu & Wang, 2019). Several studies based on nitrogen addition experiments have demonstrated that nitrogen addition can influence temporal stability through various mechanisms (Huang et al., 2020; Wu & Wang, 2019; Xu et al., 2015). Meanwhile, extreme events have the potential to cause dramatic changes in plant physiology, population dynamics, and ecosystem structure, with cascading effects on biogeochemical cycling (De Boeck et al., 2018). Multiple climate change factors simultaneously impact plant performance, community structure, and productivity, and these simultaneous changes may have the potential to interact, causing novel states of ecosystem functioning and stability (Shi et al., 2016). Moreover, different mechanisms of community stability and different ecological processes might be operating within the same community, and the stability and compensatory dynamics at different taxonomic levels may respond differently to climate change. An important goal of future stability research should be to focus on the multivariate processes of community stability, based on different taxonomic orders (Xu et al., 2015). In addition, the stability of the whole study period was significantly lower than that of the moving average (Figure 7), indicating that different climate change time scales have different impacts on community stability. Given the high spatial heterogeneity of the QTP, and the importance of stability studies on alpine grasslands not only at different temporal scales but also at different spatial scales, more long‐term studies of natural ecosystems are needed to assess the generality of our findings to other alpine grassland ecosystems.

The moving window method in our study may cause a statistical averaging effect, but it could capture lag information as plants and their communities have a lagged response to the long‐term effects of resource feedback, growth, and competition (Hu et al., 2008). We have used the piecewiseSEM to reduce error, as a relatively small number of samples were available when compared to what is generally required (i.e., 5–10 data points per path in the model). The standard coefficients greater than 1 on some paths in SEMs were caused by the small number of samples and we will continue to monitor in the future to improve the availability of data.

In conclusion, our study highlighted the importance of the dominant functional group and functional groups asynchrony for maintaining community biomass stability in alpine meadows. Furthermore, we identified a possible ‘trade‐off’ relationship between the effects of dominant functional groups and functional groups asynchrony on community biomass stability. Climate factors can directly or indirectly affect community biomass stability. Notably, the mechanisms underlying the influence of climate on community stability varied with the community composition and the local climate. Our results may offer new insight into the study of stability dynamics for alpine meadow communities under global climate change. The generality of the stability mechanism should be carefully assessed based on studies across different climate gradients and community types in the future.

## CONFLICT OF INTEREST

We declare no conflict of interest.

## AUTHOR CONTRIBUTION

**Chunyu Wang:** Conceptualization (equal); Formal analysis (equal); Investigation (equal); Methodology (equal); Software (equal); Validation (equal); Visualization (equal); Writing – original draft (equal); Writing – review & editing (equal). **Junbang Wang:** Conceptualization (equal); Funding acquisition (equal); Project administration (equal); Resources (equal); Supervision (equal); Writing – original draft (equal); Writing – review & editing (equal). **Fawei Zhang:** Conceptualization (equal); Methodology (equal); Writing – original draft (equal); Writing – review & editing (equal). **Yongsheng Yang:** Writing – original draft (equal); Writing – review & editing (equal). **Fanglin Luo:** Writing – original draft (equal); Writing – review & editing (equal). **Yingnian Li:** Funding acquisition (equal); Project administration (equal); Resources (equal); Supervision (equal); Writing – original draft (equal); Writing – review & editing (equal). **Jiexia Li:** Writing – original draft (equal); Writing – review & editing (equal).

### OPEN RESEARCH BADGES

This article has earned an Open Data Badge for making publicly available the digitally‐shareable data necessary to reproduce the reported results. The data is available at [https://doi.org/10.5061/dryad.hqbzkh1hw].

## Supporting information

Supplementary Material

## Data Availability

The data for biomass temporal stability analysis are available in Dryad (https://doi.org/10.5061/dryad.hqbzkh1hw). The biomass data and monthly meteorological data that support the findings of this study are available from the corresponding authors upon reasonable request.
