# Supplementary material for: Stability response of alpine meadow communities to temperature and precipitation changes on the Northern Tibetan Plateau
Source: Ecol Evol. 2022 Feb 16;12(2):e8592. doi: 10.1002/ece3.8592 (PMC8848471; doi:10.1002/ece3.8592)
Supplement: Supplementary file 1 — Supplementary Material [file ECE3-12-e8592-s001.docx]

Supporting Information for

**Stability response of alpine meadow communities to temperature and precipitation changes on the Northern Tibetan Plateau**

Chunyu Wang, Junbang Wang, Fawei Zhang, Yongsheng Yang, Fanglin Luo

Yingnian Li, Jiexia Li

**This file includes:** Figs. S1 to S2


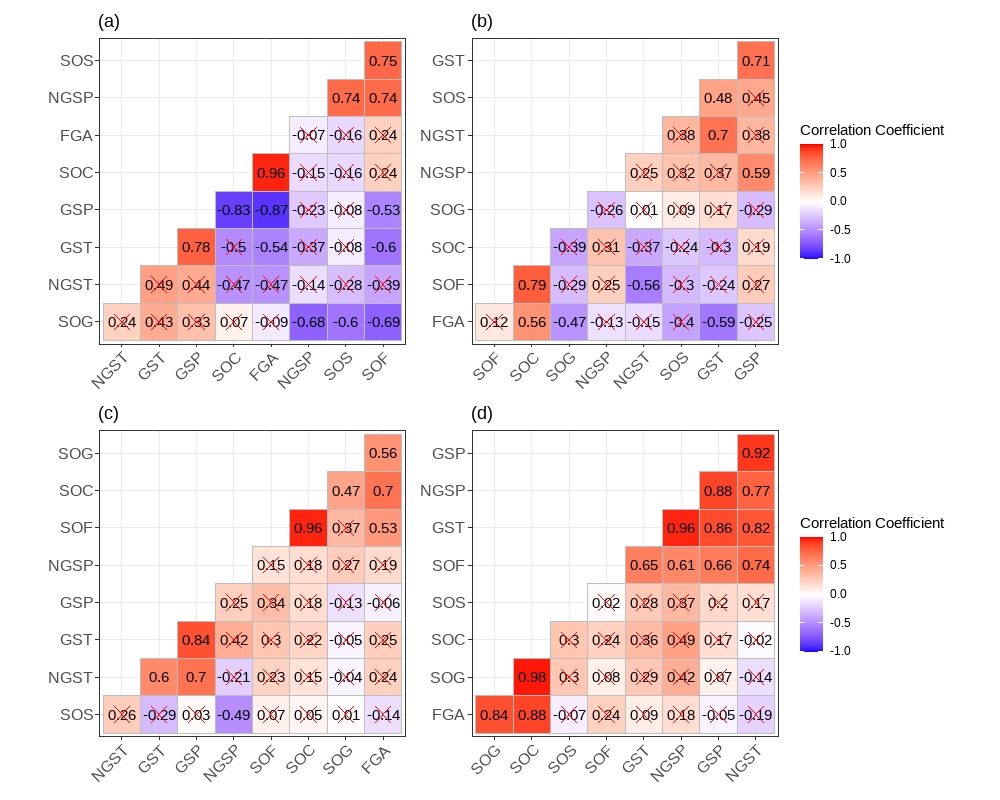


**Figure S1.** Relationships between climatic factors and biomass temporal stability of community and functional groups, and relationships between biotic and abiotic factors in (a) Haiyan, (b) Henan, (c) Gande and (d) Qumalai. The numbers in the boxes represent the Pearson correlation coefficient and the red crosses in the boxes denote the relationship with *p* ≥ 0.05

*Note*: SOG, stability of grasses; SOS, stability of sedges; SOF, stability of forbs; SOC, stability of community; FGA, functional groups asynchrony; NGST, growing season temperature; NGST, non-growing season temperature; GSP, growing season precipitation; NGSP, non-growing season precipitation.


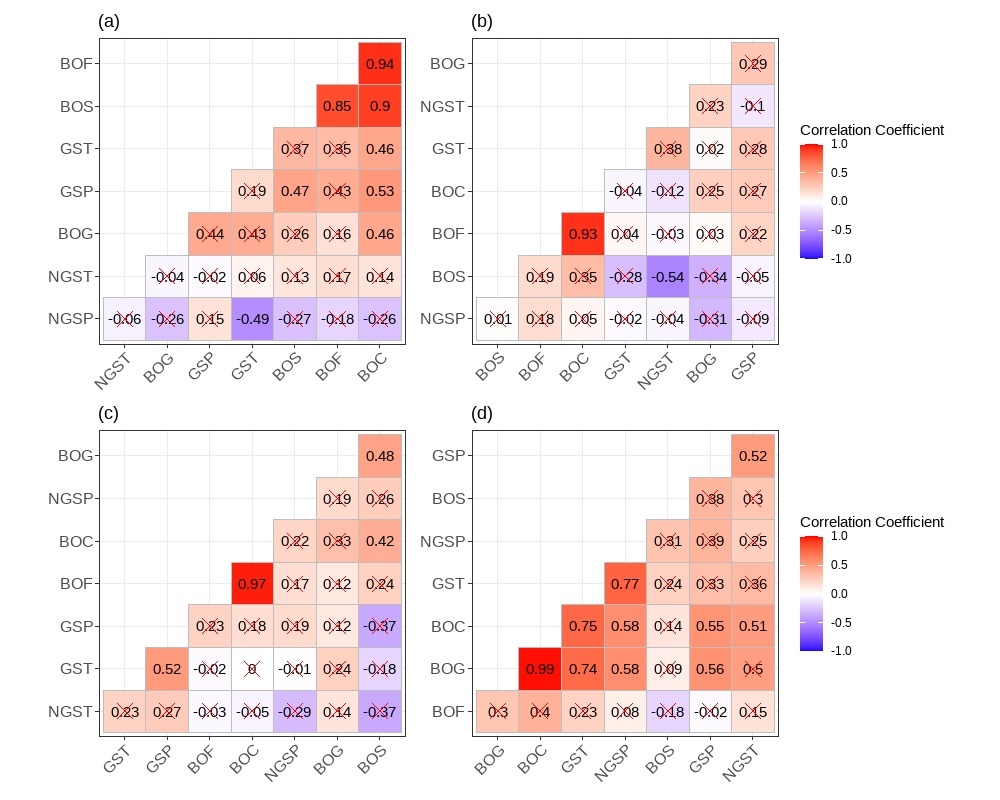


**Figure S2.** Relationships between biomass of community and functional groups and climatic factors (GST, NGST, GSP and NGSP) in (a) Haiyan, (b)Henan, (c) Gande and (d) Qumalai. The numbers in the boxes represent the Pearson correlation coefficient and the red crosses in the boxes denote the relationship with *p* ≥ 0.05.

*Note*: BOG, aboveground biomass of grasses; BOS, aboveground biomass of sedges; BOF, aboveground biomass of forbs; BOC, aboveground biomass of community; GST, growing season temperature; NGST, non-growing season temperature; GSP, growing season precipitation; NGSP, non-growing season precipitation.
